# A randomized sham-controlled trial of high-dosage accelerated intermittent theta burst rTMS in major depression: study protocol

**DOI:** 10.1186/s12888-023-05470-9

**Published:** 2024-01-08

**Authors:** Michelle S. Goodman, Fidel Vila-Rodriguez, Melanie Barwick, Matthew J. Burke, Jonathan Downar, Jonathan Hunter, Tyler S. Kaster, Yuliya Knyahnytska, Paul Kurdyak, Robert Maunder, Kevin Thorpe, Alisson P. Trevizol, Daphne Voineskos, Wei Zhang, Daniel M. Blumberger

**Affiliations:** 1 Temerty Centre for Therapeutic Brain Intervention, Centre for Addiction and Mental Health, Toronto, ON Canada; 2 Non-Invasive Neurostimulation Therapies Laboratory, Department of Psychiatry, University of British Columbia, Vancouver, BC Canada; 3 Dalla Lana School of Public Health, University of Toronto, Toronto, ON Canada; 4 The Hospital for Sick Children, Toronto, ON Canada; 5 Department of Psychiatry, University of Toronto, Toronto, ON Canada; 6 Brain Sciences Research Program, Sunnybrook Research Institute, Toronto, ON Canada; 7 Mount Sinai Hospital, Toronto, ON Canada; 8 Institute for Mental Health Policy Research, Centre for Addiction and Mental Health, Toronto, Canada; 9 Applied Health Research Centre, St. Michael’s Hospital, Toronto, ON Canada; 10 Centre for Advancing Health Outcomes, St Paul’s Hospital, Vancouver, BC Canada; 11 Faculty of Pharmaceutical Sciences, University of British Columbia, Vancouver, BC Canada

**Keywords:** Repetitive transcranial magnetic stimulation (rTMS), Accelerated intermittent theta burst stimulation (aiTBS), Treatment-resistant depression (TRD)

## Abstract

**Background:**

Intermittent theta burst stimulation (iTBS), a novel form of repetitive transcranial magnetic stimulation (rTMS), can be administered in 1/10th of the time of standard rTMS (~ 3 min vs. 37.5 min) yet achieves similar outcomes in depression. The brief nature of the iTBS protocol allows for the administration of multiple iTBS sessions per day, thus reducing the overall course length to days rather than weeks. This study aims to compare the efficacy and tolerability of active versus sham iTBS using an accelerated regimen in patients with treatment-resistant depression (TRD). As a secondary objective, we aim to assess the safety, tolerability, and treatment response to open-label low-frequency right-sided (1 Hz) stimulation using an accelerated regimen in those who do not respond to the initial week of treatment.

**Methods:**

Over three years, approximately 230 outpatients at the Centre for Addiction and Mental Health and University of British Columbia Hospital, meeting diagnostic criteria for unipolar MDD, will be recruited and randomized to a triple blind sham-controlled trial. Patients will receive five consecutive days of active or sham iTBS, administered eight times daily at 1-hour intervals, with each session delivering 600 pulses of iTBS. Those who have not achieved response by the week four follow-up visit will be offered a second course of treatment, regardless of whether they initially received active or sham stimulation.

**Discussion:**

Broader implementation of conventional iTBS is limited by the logistical demands of the current standard course consisting of 4–6 weeks of daily treatment. If our proposed accelerated iTBS protocol enables patients to achieve remission more rapidly, this would offer major benefits in terms of cost and capacity as well as the time required to achieve clinical response.

**Trial registration:**

ClinicalTrials.gov Identifier: NCT04255784.

## Background and rationale

Major depressive disorder (MDD) is a highly prevalent and disabling disorder, in which one-third of patients are classified as treatment-resistant [1]. Electroconvulsive therapy (ECT) is the most effective treatment option for those with treatment-resistant depression (TRD); however, it is used in < 1% of patients with TRD [2] due in large part to the cognitive adverse effects [3–5], need for anesthesia and societal stigma [6], associated with ECT treatment. Thus, alternative treatment approaches are urgently needed.

Repetitive transcranial magnetic stimulation (rTMS) is a safe and effective treatment for TRD that uses powerful, focused magnetic field pulses, applied non-invasively, to induce lasting changes in the activity of brain regions involved in regulating thoughts, emotions, and behaviour [7–9]. Across several studies [10, 11] rTMS has demonstrated high response and remission rates of up to 50% and 35%, respectively. The current standard rTMS treatment protocol involves applying 10 Hz stimulation daily to the left dorsolateral prefrontal cortex (DLPFC), over 37.5 min. [12]. Additionally, average treatment courses last between 4 and 6 weeks, given that current studies suggest the effects of rTMS treatment are linearly cumulative [13–15], with maximal benefits after 25 to 28 treatments [14, 16, 17]. These long treatment sessions and courses result in two major drawbacks to rTMS: high cost and low capacity.

Intermittent theta burst stimulation (iTBS), a novel form of rTMS, addresses these key issues as treatment can be administered in 1/10th of the time of standard high frequency rTMS (~ 3 min vs. 37.5 min). Importantly, iTBS has resulted in similar or greater effects on neural plasticity [18] and clinical outcomes [19]. Major gains in rTMS efficiency and accessibility could be realized by administering multiple iTBS sessions per day, thus significantly reducing course length. Several recently published studies suggest that accelerated rTMS may be feasible, tolerable, and capable of achieving comparable remission rates to standard rTMS in shorter time-frames of 4 to 10 days [20–25]. However, the majority of these studies were small, open-label case series that did not control for the non-specific effects of multiple daily interventions.

## Objectives

The primary objective of this study is to compare the efficacy and tolerability of active versus sham iTBS using an accelerated regimen of 8 daily sessions administered at 1-hour intervals, for five days, in patients with TRD. As a secondary objective, we aim to assess the safety, tolerability and treatment response to open-label low-frequency right-sided (1 Hz) stimulation using an accelerated regimen of 8 daily sessions for five days, in patients with TRD who do not respond to an initial week of blinded left DLPFC iTBS stimulation.

## Methods

### Trial design and setting

Over three years, approximately 230 outpatients at CAMH and the University of British Columbia Hospital, meeting diagnostic criteria for MDD, will be recruited and randomized to a triple-blind sham-controlled trial (patient, rater and technician blinding). Combined, these two centres receive 600–800 referrals annually for psychiatric brain stimulation from nurse practitioners, family physicians and psychiatrists. At the CAMH site, referrals are received from across the province of Ontario for patients from all genders and across the adult lifespan. A similarly diverse population are referred to the UBC site, from metro Vancouver areas. We believe that this recruitment approach will lead to the inclusion of a wide range of patients with diverse backgrounds.

**Fig. 1:**
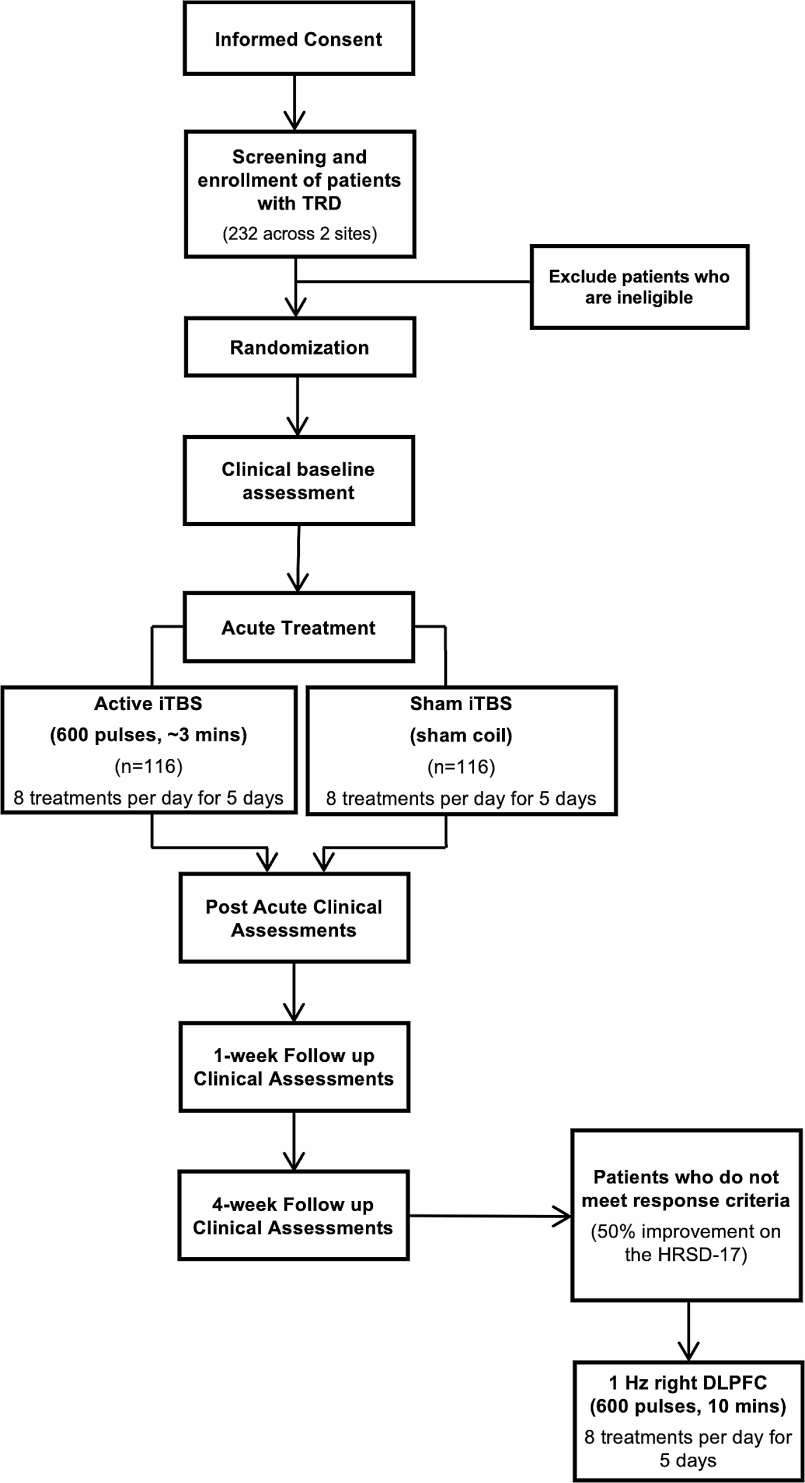
Study flow chart

Patients will receive five consecutive days (Monday to Friday) of active or sham iTBS, administered eight times daily at 1-hour intervals, with each session delivering 600 pulses of iTBS over 3 min and 9 s. Sham treatments will be delivered using a shielded “sham coil” that reproduces auditory and tactile sensations of stimulation. Refer to Fig. 1 for a summary of the trial design.

Following randomization, patients will undergo a series of assessments and motor threshold testing to determine the appropriate strength of stimulation. Depressive symptoms will be assessed using the Hamilton Depression Rating Scale (HRSD-17) [26]. The primary outcome analysis will include the change in HRSD scores from baseline to the end of the acute treatment. The HRSD-17 was selected to facilitate the comparison of outcomes in this trial to that of previous trials, since the HRSD has been standard in both rTMS and pharmacotherapy trials of patients with depression for decades [27–31]. The HRSD-6 [32], derived from the HRSD-17, will also be included as a secondary outcome measure. The Scale for Suicide Ideation (SSI) and self-report scales of depression and anxiety (Beck Depression Inventory, BDI-II and the General Anxiety Disorder, GAD-7), will also be completed. Side effects will be recorded at every visit and after every treatment session. Final assessments will be completed immediately after the last treatment. The one-week and four-week follow up assessments will be allowed within 1–2 days of the scheduled time points. Refer to Table 1 for a detailed schedule of assessments.

All treatments will be conducted with minimized personal contact (i.e., verbal communication) between the technician and patient to reduce the impact of nonspecific therapeutic contact on outcomes. The relevance of alliance extends beyond psychotherapy and has been shown to impact pharmacological interventions [33]. As a way to quantify the nonspecific active component of the treatment, a modified Human Connections Scale (HCS) will be administered following each treatment course (blinded and open label) to determine the impact of the patient’s sense of alliance with the technician on the therapeutic outcome [34]. This scale will be used as a secondary measure and explored with respect to its role in the treatment outcome.

Open Label: To ensure that all patients can receive a form of active treatment, those who have not achieved the response criterion (i.e., a 50% improvement from baseline on the HRSD-17) at the week four follow-up visit will be offered a second course of treatment, regardless of whether they initially received active or sham treatment.

The blind will be maintained, and no further assessment contributing to the primary hypotheses will occur after the 4-week time point. A different technician will administer the open-label second course of treatment so as not to unblind the technician to the original treatment course. To ensure that patients do not simply receive the same treatment pattern that previously failed to exert an adequate effect, the second course of treatment will apply active rTMS using low-frequency (1 Hz) stimulation for 600 pulses (10 min), 8 times daily at 1-hour intervals for five days. 1 Hz right-sided rTMS has been shown in large-scale trials to achieve outcomes similar to left high-frequency stimulation [35], which has equivalent outcomes to iTBS [19]. All patients completing the second course of treatment will undergo the same schedule of clinical assessments during and after the course of treatment. Should the participant be available to start the second course of treatment immediately, the four-week follow-up assessment from the first course of treatment will serve as the baseline for the unblinded 1 Hz treatment. Those who go on to receive the second treatment course but cannot attend treatment immediately, will have a separate baseline assessment. Participants will be allowed to start the open-label course of treatment up to 3 months after the 4-week follow-up of the first course. Female participants will complete an abbreviated reproductive history immediately before the second course of treatment.

### Eligibility criteria

Inclusion Criteria: Patients will be included if they: (1) are outpatients between the ages of 18 and 65; (2) are voluntary and competent to consent to treatment; (3) have a Mini-International Neuropsychiatric Interview (MINI) confirmed diagnosis of MDD, single or recurrent; (4) have failed to achieve a clinical response to an adequate dose of an antidepressant based on an Antidepressant Treatment History Form (ATHF) score for that antidepressant trial of ≥ 3 in the current episode [36, 37] OR have been unable to tolerate at least 2 separate trials of antidepressants of inadequate dose and duration (ATHF score of 1 or 2 on those 2 separate antidepressants) in the current episode; (5) have a minimum of moderate depression as indicated by a score of > 9 on the PHQ-9, in order to separate inclusion criteria from the primary outcome assessment; (6) have had no increase or initiation of any antidepressant or augmentation medication in the 4 weeks prior to screening; (7) able to adhere to the treatment schedule; (8) Pass the TMS adult safety screening (TASS) questionnaire; (9) have normal thyroid functioning based on pre-study blood work.

Exclusion Criteria: Patients are excluded if they: (1) have a MINI confirmed diagnosis of substance dependence or abuse within the last 3 months; (2) have a concomitant major unstable medical illness, cardiac pacemaker or implanted medication pump; (3) have active suicidal intent; (4) are pregnant; (5) have a lifetime MINI diagnosis of bipolar I or II disorder, schizophrenia, schizoaffective disorder, schizophreniform disorder, delusional disorder, or current psychotic symptoms; (6) have a MINI diagnosis of obsessive compulsive disorder, post-traumatic stress disorder (current or within the last year), anxiety disorder (generalized anxiety disorder, social anxiety disorder, panic disorder), or dysthymia, that is assessed by a study investigator to be primary and causing greater impairment than MDD; (7) have a diagnosis of any personality disorder, assessed by a study investigator to be primary and causing greater impairment than MDD; (8) have failed a course of ECT in the current episode or previous episode; (9) have received rTMS for any previous indication due to the potential compromise of subject blinding; (10) have any significant neurological disorder or insult including, (e.g., any condition likely associated with increased intracranial pressure, space occupying lesion, history of seizure except those therapeutically induced by ECT or a febrile seizure of infancy, cerebral aneurysm, Parkinson’s disease, Huntington’s chorea, multiple sclerosis, significant head trauma with clear radiological evidence of cerebrovascular injury on imaging); (11) have an intracranial implant (e.g., aneurysm clips, shunts, stimulators, cochlear implants, or electrodes) or any other metal object within or near the head, excluding the mouth, that cannot be safely removed; (12) if participating in psychotherapy, must have been in stable treatment for at least 3 months prior to entry into the study, with no anticipation of change in the frequency of therapeutic sessions, or the therapeutic focus over the duration of the study; (13) clinically significant laboratory abnormality, in the opinion of the one of the principal investigators or study physicians; (14) currently take more than lorazepam 2 mg daily (or equivalent) or any dose of an anticonvulsant due to the potential to limit rTMS efficacy.

### Informed consent procedure

The informed consent process is initiated before the patient agrees to participate in the study and will be obtained according to REB and GCP guidelines. All patients referred by a general physician or psychiatrist will undergo an extensive consultation with a brain stimulation psychiatrist at each study site, who will determine suitability for referral to this trial. Patients referred to the trial will then be assessed for eligibility by qualified research personnel who will obtain consent. Patients will be informed that they can withdraw participation at any point during the study, and the rights and welfare of the patients will be protected.

### Randomization and blinding

Participants will be randomized into the study, stratified by site and by medication resistance (2 or fewer adequate trial failures versus more than 2 adequate trial failures; an adequate trial of antidepressant is defined as an antidepressant trial ranked with an ATHF (score ≥ 3)). Prior rTMS trials have demonstrated that the degree of treatment resistance is a key predictor of response, and therefore, it is essential to ensure that the groups are balanced concerning this variable [12, 38, 39].

Patients will be randomized using random permuted blocks of varying sizes, with study personnel blinded to the block sizes. An independent assistant external to the study will manage the randomization using a computer generator. The participants’ number and treatment code will be assigned after their details (i.e., initials and number of failed adequate antidepressant trials) have been obtained. A unique treatment code, designated to either active or sham stimulation, allows for the technicians to remain blinded. Once the code is entered into the machine, it indicates which side of the A/P coil to place over the stimulation site and will only deliver pulses when the designated coil is placed correctly.

A specially designed rTMS coil containing both an active and a sham inductor head and identical external appearance will be used to ensure blinding during treatment. A computerized sensor instructs the technician to rotate the coil so that one side is in contact with the scalp. A pair of electrodes will also be placed close to the treatment spot, directly on the forehead, 1 cm apart from each other and secured with medical tape, in both active and sham groups. In the sham group, electrical pulses will be delivered through the scalp electrodes on each TMS pulse to further mimic the scalp sensations and muscle contractions associated with *verum* TMS. This approach is most likely to achieve both patient and technician blinding and is seen as the best possible sham for blinded rTMS treatment trials [24, 40].

An independent, blinded rater at each site will administer the clinical assessment scales the week before and after the final treatment and at four weeks post-treatment. At baseline, participants will be asked about their expectancy of improving following the treatment course using the Stanford Expectancy Scale [41]. In addition, to assess the integrity of blinding, after their first treatment day and at the 4-week follow-up (for participants)/1-week follow-up (for technicians), all patients and technicians will be asked whether or not they believe the active or sham treatment was delivered along with their degree of certainty (0 guess – 10 certain). Collecting this information will help determine the extent of underlying placebo or nocebo effects concerning their expectations and therapeutic outcomes [42, 43].

### Intervention

#### rTMS treatment parameters

rTMS will employ the MagPro X100/R30 stimulator (MagVenture, Farum, Denmark) equipped with the B70 A/P fluid-cooled coil, which has both an active and a placebo head actuated under computer control to maintain blinding. The modified BeamF3 scalp heuristic will be used to localize the treatment site over the left DLPFC [44].

Before the first treatment, each participant’s motor threshold will be determined according to published methods [45, 46]. This location and the stimulation target site will be marked at the first session on the scalp, and standard methods will be used to target this site during treatment. All patients will undergo 8 treatment sessions per day for 5 consecutive days, with the start of each session timed to be at least 50 min from the previous session. While the optimal time interval between treatment sessions remains unknown, longer intersession intervals (ISI), of approximately 1 h and above, are suggested to induce cumulative effect on synaptic strengthening compared to shorter ISIs [47–50]. Additionally, this intersession interval was chosen to be line with previously published high dose aiTBS trials [25, 51]. Each session will deliver 600 pulses of iTBS (bursts of 3 pulses at 50 Hz, bursts repeated at 5 Hz, with a duty cycle of 2 s on, 8 s off, over 60 cycles / ~3 min) at a target of 120% of the participant’s resting motor threshold. Based on their tolerability, intensity can be decreased to a minimum of 90%. We selected 600 pulses for each session, given recent research suggesting that 600 vs. 1800 pulses of TBS may have similar neurophysiological [52] and clinical [53] effects.

For those undergoing the second course of treatment, rTMS will be delivered with an active, B70 fluid-cooled coil over the right DLPFC located using the scalp heuristic above for the F4 electrode. The schedule of treatments will remain the same: 600 pulses of 1 Hz stimulation over 10 min and 8 sessions per day for 5 consecutive days, with the start of each session timed to be at least 50 min from the previous session.

#### rTMS treatment side effects/risks

Many thousands of people have received rTMS treatment over the last 20 years. rTMS has certain risks; while some of these risks are known, there is a possibility of risks that we do not know about and have not been seen in study participants to date.

rTMS is recognized in the most recent consensus safety guidelines as a safe and well-tolerated treatment for the vast majority of individuals [54], with an all-causes dropout rate several-fold lower than for antidepressant medications [55]. Common and rare but serious risks are described as below (the numbers in brackets show how often the side-effect happened.):

1. Common: Headache (30%), discomfort or pain at the stimulation site (20%), lightheadedness or dizziness after the treatment (20%), facial muscle twitching (30%). These side effects are mild, generally diminish over treatment, and can usually be managed with rest or over-the-counter pain medications such as acetaminophen or ibuprofen.
2. Less common: (1–7%) fatigue, headache persisting after treatment, dizziness or fainting during the initial sessions of rTMS treatment.
3. Rare but serious: Onset of suicidal thinking (< 1%); hypomanic episode (< 1%).
4. Very rare but serious: There are rare cases of an epileptic seizure resulting from rTMS (less than 0.1%) [56]. Safety guidelines have been in place since 1997 to minimize the risk of seizures from rTMS, and this study follows those guidelines.

Common side effects of rTMS treatment are expected and will be recorded separately from adverse events. Participants will be asked to defer any changes to their antidepressant medications for four weeks before and during the course of rTMS to avoid confounding effects. A numeric rating scale (0 no pain – 10 worst pain they have experienced) will be administered at every visit and after every treatment session, to rate the severity of pain from side effects along with one open-ended question asking about other side effects. The technician will complete these questions so that side effect severity and resolution can be assessed and verified appropriately over time.

### Schedule of events

#### Screening evaluation

Patients will be assessed using the MINI to assess current and lifetime depression and other psychiatric disorders, and will be used to verify psychiatric inclusion. The screening information based on the current episode, will be determined from the patient report and the records of a pre-study clinical assessment, conducted by a psychiatrist trained in TRD. The ATHF is a commonly used and reliable method of assessing the adequacy of prior antidepressant treatment [36, 37] and will be used to confirm eligibility based on inclusion criteria for treatment resistance. To address the potential for rater bias, whereby baseline scores are inflated to ensure patient eligibility [57], we will employ two separate depression rating scales, one to determine eligibility and a different scale to serve as the primary outcome measure [58]. As such, we will require a minimum severity of moderate depression on a separate measure, the patient health questionnaire (PHQ-9 > 9) [59], as an inclusion criterion. The Transcranial Magnetic Stimulation Adult Safety Screen (TASS) will be used to assess potential rTMS risk factors. A pre-study blood test that includes electrolytes, complete blood count, and thyroid stimulating hormone will be required to rule out any underlying medical causes of the depression. These results will be accepted if completed within the lesser of the current depressive episode or six months.

#### Clinical assessments during and after treatment

Clinical measures will be assessed the week before treatment and immediately after treatment completion (Friday, Visit 5), as well as at 1 and 4 weeks after treatment completion. The schedule of assessments is located in Table 1.

To compare results to other depression studies, we will examine depressive symptoms using clinician-rated and self-report scales. Secondary outcome measures will include the HRSD-6, SSI, the self-rated BDI-II, and the GAD-7. Female participants will also complete a complete reproductive history the week before treatment since the effects of high-frequency rTMS have been shown to vary based on circulating hormones [60].

**Table 1:** Schedule of Assessments

Clinical Assessment Scales*	Screening and Baseline	Treatment Course 1		Follow-up Period 1			Treatment Course 2		Follow-up Period 2	
	Week 0	Visit 1,2, 3,4	Visit 5	Week 1	Week 4		Visit 1,2, 3,4	Visit 5	Week 1	Week 4
MINI	X									
TASS	X									
GAD-7	X		X	X	X			X	X	X
PHQ-9	X									
SSI	X		X	X	X			X	X	X
HRSD-17	X		X	X	X			X	X	X
HRSD-6	X		X	X	X			X	X	X
ATHF	X									
BDI-II	X	X	X	X	X		X	X		X
Side effect report	X	X	X	X	X		X	X	X	X
Reproductive history (females only)	X						X (prior to Visit 1 only)			
Substance Use Questions	X	X	X				X	X		
Stanford Expectancy Questionnaire	X						X (prior to Visit 1 only)			
HCS				X					X	
Group Assignment (participants and technicians)		X (after visit 1)		X	X					

MINI: Mini-International Neuropsychiatric Interview; TASS: Transcranial Magnetic Stimulation Adult Safety Screen; GAD-7: Generalized Anxiety Disorder 7-Item; PHQ-9: Patient Health Questionnaire 9-Item; SSI: Scale for Suicidal Ideation; HRSD-17: 17 Item Hamilton Rating Scale for Depression; HRSD-6: 6 Item Hamilton Rating Scale for Depression; ATHF: Antidepressant Treatment History Form; BDI-II: Beck Depression Inventory; HCS: Human Connections Scale

### Attendance and withdrawal criteria

Patients will be encouraged to attend all scheduled treatments. Those that meet the following criteria will be excluded from the per protocol analysis if they:

1. Miss / fail to attend any one of the five treatment days in the course overall.
2. Miss / fail to attend more than five treatment sessions over the five days.
3. Cannot tolerate stimulation of at least 90% RMT for the entire session on more than five treatment sessions.
4. Develop active suicidal ideation with intent during the course of stimulation, or in the opinion of the site PI, that participation is not clinically indicated.
5. Are admitted to hospital during the course of treatment.
6. Withdraw consent to participate.

Patients will be discontinued if they experience worsening in depression, defined as an increase in HRSD-17 from a baseline of more than 25% at the post-treatment assessment or development of active suicidal intent or attempted suicide.

### Sample size

We will consider the 8 × 5 protocol successful if the active stimulation group achieves superior improvement (i.e., a larger reduction in the mean HRSD-17 score) versus sham on the HRSD-17. To detect the minimally clinically significant difference of three points [61] on the HRSD-17 with 80% power (based on post-treatment HRSD-17 standard deviation of 8 and pre-post correlation of 0.27 in the iTBS group in the THREE-D trial [19]), 105 patients per group are needed. We expect a low attrition of 10% given that the treatment course is only five days, increasing the number required to be randomized to 116 per group (232 in total).

### Statistical methods

#### Clinical outcomes analysis

Primary Outcome Analysis: The primary outcome analysis on the change in HRSD-17 between baseline and end of treatment, will be conducted on an intention-to-treat basis. We plan to use multiple imputation methods developed by Schafer [62] to account for potential bias that may be incurred by missing data. Before the main effects analysis, descriptive statistics will be generated to summarize the data on all randomized participants to confirm that there are no group differences between the two conditions concerning baseline demographics and clinical characteristics. To assess for significant differences in treatment effects over time, a mixed-effects model with predictor variables for group (active vs. sham), time (baseline vs. end of treatment), and group-by-time interaction will be fitted to the data from the primary outcome measure (HRSD-17). The mixed-effects model will also be used to estimate the treatment effect over the full course of treatment.

Secondary Outcome Analyses: We will repeat the primary and secondary analyses in the same manner for the one-week post-treatment and four-week post-treatment time points. The same approach will be employed to assess treatment effects on the HRSD-6, SSI, BDI-II and GAD-7. Response and remission will be included as secondary clinical outcomes. Response will be defined as ≥ 50% reduction in symptoms on the primary outcome measure (HRSD-17) and remission will be defined as a post-treatment HRSD-17 < 8 [1]. We will use a conservative imputation that assumes non-remission or non-response if data is missing at the post-treatment assessment. A two-tailed Chi-squared test will assess the significance of any observed differences in the proportion of responders and remitters between groups based on pre- to post-treatment HRSD-17. Finally, we will summarize and compare the rates of adverse events, severe adverse events and dropouts in both groups.

Exploratory Outcome Analyses: A multivariable regression model will be used to explore whether or not treatment resistance, patient expectancy, patient experience, or HCS modify the baseline adjusted treatment effect on the HRSD-17 (at day 5). The same analytic approach used for the primary outcome will be employed to assess for treatment effects on the HRSD-6, BDI-II, SSI and GAD-7. We will descriptively report the change from baseline to 5 days, 1-week, and 4-week post-treatment in the open-label 1 Hz right DLPFC phase and compare change, response, and remission in those who received active compared to sham stimulation in the blinded phase. Additionally, we will build a multiple regression model to explore how prior treatment condition, expectancy, and HCS score impact the baseline adjusted treatment effect in the open-label 1 Hz right DLPFC treatment group.

## Discussion

Broader implementation of rTMS is limited by logistical demands associated with the current standard 4–6-week course of daily treatment. This can be a significant barrier for patients who live farther from a treatment centre or those who can work or want to reduce the length of their medical leave. If the treatment regimen can be accelerated by administering multiple sessions per day, this would allow outpatients to undergo treatment with less disruption of daily activities and ultimately improve access to rTMS. This may also position rTMS as an option for patients with a need for rapid treatment effects, potentially allowing for remission in days rather than weeks. As such, there is a strong rationale to study the efficacy, safety, and tolerability of accelerated iTBS in patients with TRD.

Importantly, if our accelerated iTBS protocol demonstrates superiority over sham stimulation, it could be rapidly integrated into clinical practice with current equipment and minor modifications to clinic scheduling. Additionally, through strict adherence to blinding procedures, an advanced sham rTMS technique, and an assessment of patient expectancy and patient-technician relationship, this proposed study has the potential to provide significant insights into the role of non-specific TMS effects on clinical outcomes [43]. This large-scale, multi-site randomized control trial may broaden the use of neurostimulation by demonstrating the efficacy of a new rapid-acting treatment for this challenging, common, and burdensome illness.

### Trial status

The study is currently recruiting participants. This trial began recruitment in February 2020.

## Data Availability

The final dataset generated from the current protocol will be available from the corresponding author upon reasonable request.

## Abbreviations

aiTBS: accelerated intermittent theta burst stimulation
ATHF: Antidepressant Treatment History Form
BDI-II: Beck’s Depression Inventory
CAMH: Centre for Addiction and mental Health
DLPFC: dorsolateral prefrontal cortex
eCRF: electronic case report forms
ECT: Electroconvulsive therapy
GAD-7: Generalized Anxiety Disorder 7-Item
HRSD: Hamilton Rating Scale for Depression
ITT: intention to treat
MDD: Major depressive disorder
MINI: Mini-International Neuropsychiatric Interview
PHQ-9: Patient Health Questionnaire
rTMS: repetitive transcranial magnetic stimulation
SSI: Scale of Suicidal Ideation
TASS: Transcranial Magnetic Stimulation Adult Safety Screen
TRD: treatment-resistant depression
